# The pedicled sartorius flap and mesh (PSM) technique vs no reconstruction in repairing the defect after type III pelvic bone tumor resection: a retrospective study

**DOI:** 10.1186/s12957-023-02905-1

**Published:** 2023-01-18

**Authors:** Xinhui Du, Hua Wei, Boya Zhang, Shilei Gao, Zhehuang Li, Weitao Yao

**Affiliations:** 1 Bone and Soft Tissue Department, The Affiliated Cancer Hospital of Zhengzhou University & Henan Cancer Hospital, Zhengzhou, 450008 China; 2 Key Laboratory for Digital Assessment of Spinal-Pelvic Tumor and Surgical Aid Tools Design (Zhengzhou), 127 DongMing Road, JinShui District, ZhengZhou, 450008 HeNan China; 3 Key Laboratory for Perioperative Digital Assessment of Bone Tumors (Henan), 127 DongMing Road, JinShui District, Zhengzhou, 450008 Henan China; 4 Department of Anesthesiology, Pain and Perioperative Medicine, The First Affiliated Hospital of Zhengzhou University, 1 East JianShe Road, Zhongyuan District, Zhengzhou, 450052 Henan China

**Keywords:** Hernia, Orthopedics, Oncology, Infection

## Abstract

**Background:**

Type III pelvic bone tumor resections are often accompanied by postoperative complications. In order to reduce complications, we developed a novel pedicled sartorius flap and mesh (PSM) technique to reconstruct the pelvic ring defect. In this study, we evaluated the efficacy and risks of this PSM technique in type III pelvic bone tumor resections by comparing outcomes between patients that underwent PSM reconstruction and patients that did not receive any reconstruction.

**Methods:**

We retrospectively reviewed a consecutive set of patients that underwent type III pelvic bone tumor surgeries in our center from January 2020 to January 2021 with either PSM reconstruction (designated as the PSM group) or without any reconstruction (designated as the control group). General information such as age, gender, tumor type, tumor size, and surgical-related information such as duration of surgery, blood loss, and the surgical margins was collected. Outcome data recorded included wound complications such as infection and dehiscence, local recurrence, and Musculoskeletal Tumor Society (MSTS) scores for postoperative functional evaluation. Statistical analysis between both groups was performed with GraphPad Prism v7.

**Results:**

A total of 20 patients were included in this study (PSM group *n* = 12, control group *n* = 8). While no herniation was found in the PSM group, it occurred in 6 of 8 cases in the control group. The control group showed a significantly higher rate of bacterial infection (*p* = 0.03) and wound dehiscence (*p* = 0.02) but lower MSTS scores (*p* < 0.05) compared to the PSM group.

**Conclusions:**

The use of the PSM technique can significantly reduce postoperative complication rates and enhance postoperative function following type III pelvic bone tumor resection.

## Background

Pelvic bones are a common location for both primary and metastatic tumors [1, 2]. Despite recent developments in novel pharmacological therapies and radiotherapy, surgical resection remains the standard of care for localized diseases [3, 4].

Enneking et al. proposed a location-based approach to reconstructing lesions following pelvic bone tumor resection [5]. For tumors arising in weight-bearing regions such as the acetabulum and sacroiliac joint, reconstruction was often accomplished through allografts, autografts, or more recently 3D-printed personalized prostheses [3, 6–8]. However, reconstruction of defects left by pelvic tumor resection is associated with high rates of complications such as dislocation, deep venous thrombosis, and prosthetic loosening or fracture [9–11]. In certain situations, tumor resection without any reconstruction has even been reported to result in better outcomes than if reconstruction was performed [12].

Resection of type III pelvic bone tumors involves the resection of the pubis or/and ischium based on the Enneking classification, typically resulting in massive structural loss and dead space. Multiple reconstructive methods have been proposed to accompany type III resections, such as allografts and 3D-printed customized prostheses [13]. However, graft displacement, herniation, and several other complications tend to compromise overall outcomes [14, 15].

In order to deal with pelvic ring defects and reduce complications following type III resections, we developed a novel technique of pedicled sartorius flap and mesh (PSM) to reconstruct the defect and fill the dead space. In this study, we aimed to assess the efficacy and risks of the PSM technique in reconstructing bone defects and reducing perioperative complication rates following type III pelvic resection. We retrospectively analyzed a consecutive set of patients in our center undergoing type III pelvic resection reconstructed with either PSM technique or no reconstruction. We found that PSM reconstruction can significantly reduce perioperative complication rates and enhance postoperative functions.

## Methods

We retrospectively reviewed consecutive patients that underwent a type III pelvic bone tumor resection in our institution from January 2020 to January 2021. Inclusion criteria: (1) patient age of 16–80 years old, (2) aggressive or malignant tumor affecting the ischiatic and/or pubic bone, (3) treated in our center with en bloc resection without any reconstruction or repaired with the PSM technique. Exclusion criteria: (1) prior history of radiotherapy of the pelvic region, (2) skin ulceration or infection of any kind before surgery, (3) pelvic bone tumor treated with intralesional curettage surgeries, (4) patients with diabetes mellitus or other chronic diseases requiring management with drugs that are known to affect wound healing.

The pelvic bone tumors were evaluated with the CT/MRI fusion image 3D models and surgical planes were designed based on the digital models. The resections were either guided with 3D print resection guides or navigation systems. The suspected margins were sent for intra-operative pathological examination and the margins of all resected specimens were assessed routinely by pathologists after surgery. The bone defects were either reconstructed with the PSM technique (designated as the PSM group) or left unattended (designated as the control group, without any reconstruction, not even a mesh or any other methods).

Patient information that was collected included: gender, age, location of the lesion, tumor type, longest dimension of the tumor, duration of the surgery, intraoperative blood loss, surgical margins, the use of 24 h prophylactic antibiotics, detection of bacteria, wound dehiscence, and debridement. Presence of the bacteria in wound discharge was screened through plate culture. Patients received perioperative prophylaxis of 1.5 g of cefuroxime within 24 h of the surgery (clindamycin was used in the case of allergies). In addition, we collected the follow-up information of the patients and recorded the presence of local recurrence, herniation, and functional results as assessed using the Musculoskeletal Tumor Society (MSTS) scoring system.

From January 2020 to January 2021, in our center, 20 patients underwent type III pelvic bone tumor resections. Of the 20 patients, 12 belonged to the PSM group while 8 belonged to the control group. The median age of the PSM group was 39.5 years (range 37–78) compared with 49 years (range 31–73) in the control group. The median follow-up duration was 13.5 months (range 8–19) and 15 months (range 10–19) for the PSM group and the control group, respectively. The location of the lesion in both groups was either pubis or ischium and the pathological diagnosis of this group of cases was either chondrosarcoma, giant cell tumor, or metastatic lesion.

For between-group comparisons of normally distributed variables, the p-value was calculated with unpaired Student t- tests with an alpha of 0.05. The categorical data between different groups was statistically analyzed with a chi-square test (*X*^2^) with an alpha of 0.05. All the statistical analysis was conducted with GraphPad Prism v7.

## Results

### Clinical parameters of the patients

The clinical features of the patients included in this study are summarized in Table 1. There was no significant difference in age (*p* = 0.48), follow-up duration (*p* = 0.31), gender (*p* = 0.14), location of the lesion (*p* = 0.16), or tumor type (*p* = 0.29) between the two groups. The tumor size between both groups (described as the largest dimension) was similar (*p* = 0.77).

**Table 1:** Study population characteristics

Patient characteristics	PSM group	Control group	
Total	12	8	*P* = 0.14
Male	5	3	
Female	7	5	
Age (years, median, range)	39.5 (37–78)	49 (31–73)	*P* = 0.48
Follow-up (months, median, range)	13.5 (8–19)	15 (10–19)	*P* = 0.31
Largest dimension (cm)	14.7 (9.4–21.2)	15.1 (8.9–21.5)	*P* = 0.77
Location of lesion			
Pubis	9	6	*P* = 0.16
Ischium	3	2	
Tumor type			
Chondrosarcoma	9	6	*P* = 0.29
Giant cell tumor	2	1	
Metastatic lesion	1	1	

### Case presentation and illustration of the PSM technique

**Fig. 1:**
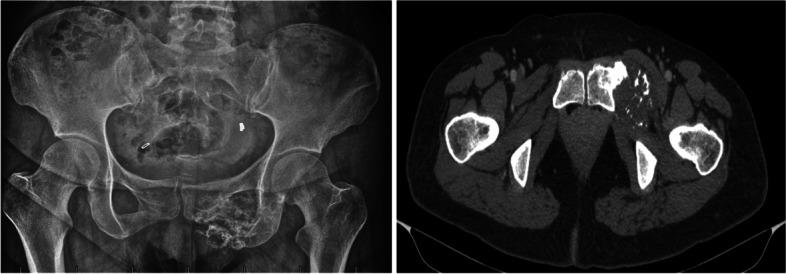
Preoperative images of a 46-year-old chondrosarcoma patient. X-ray (left) shows an irregularly-shaped lesion with calcification in the pubis region. CT scan of the pelvic bone (right) shows a tumor extending from the pubis toward the ischium with intralesional “popcorn-like” calcification

**Fig. 2:**
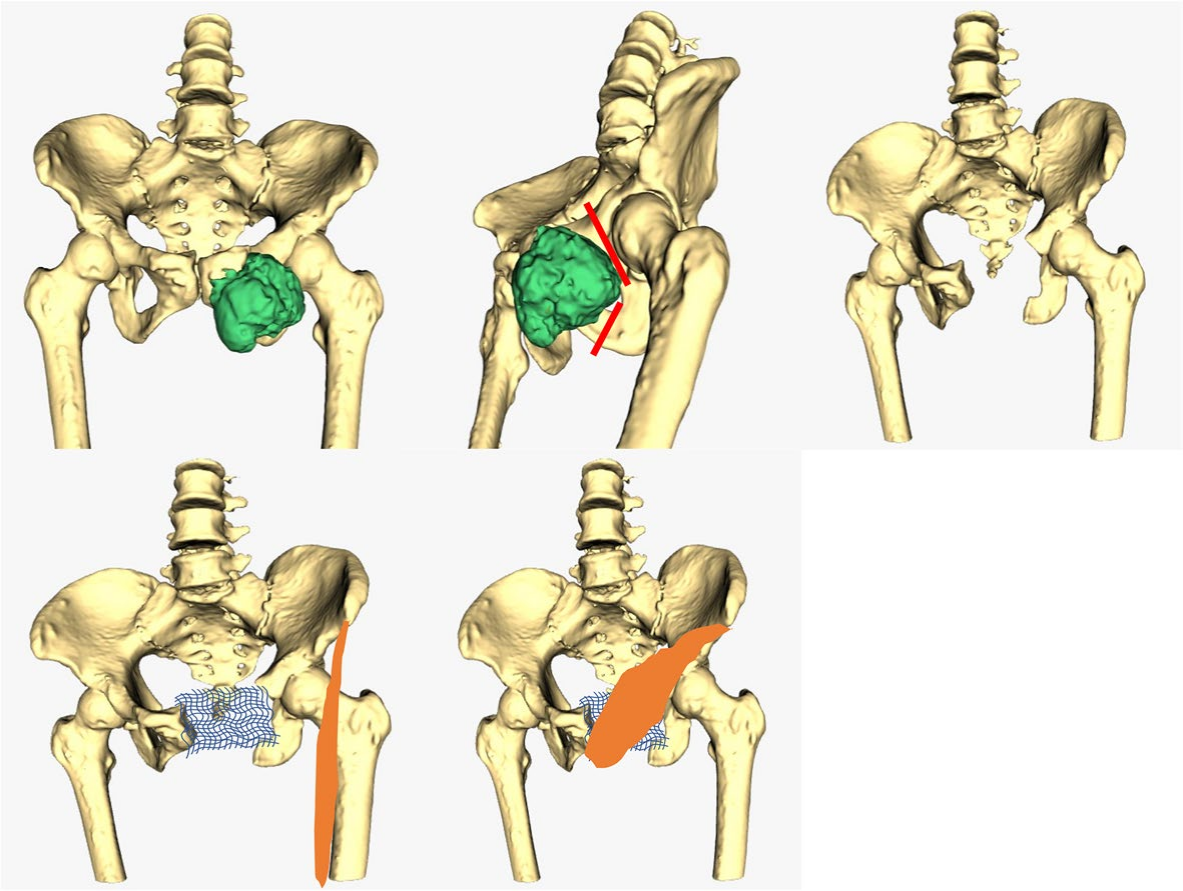
Preoperative planning involved the creation of a CT/MRI fusion 3D model of the bone tumor and affected pubis. The model was used to determine the bone resection plane (orange linear shapes)

A 45-year-old female patient was referred to our hospital with the chief complaint of a painless mass in the left groin. X-ray and CT scan (Fig. 1) were performed and a neoplasm originating from the pubis presenting with intralesional “popcorn-like” calcification was found. A core needle biopsy was conducted which suggested a diagnosis of chondrosarcoma. A CT/MRI fusion 3D model (Fig. 2) was created and surgical planes were planned based on the model as previously reported [16, 17].

#### Tumor resection and margin evaluation

The tumor was removed according to the preplanned resection plane (Fig. 2), and margins were evaluated intraoperatively and postoperatively by experienced pathologists.

#### The PSM reconstruction

**Fig. 3:**
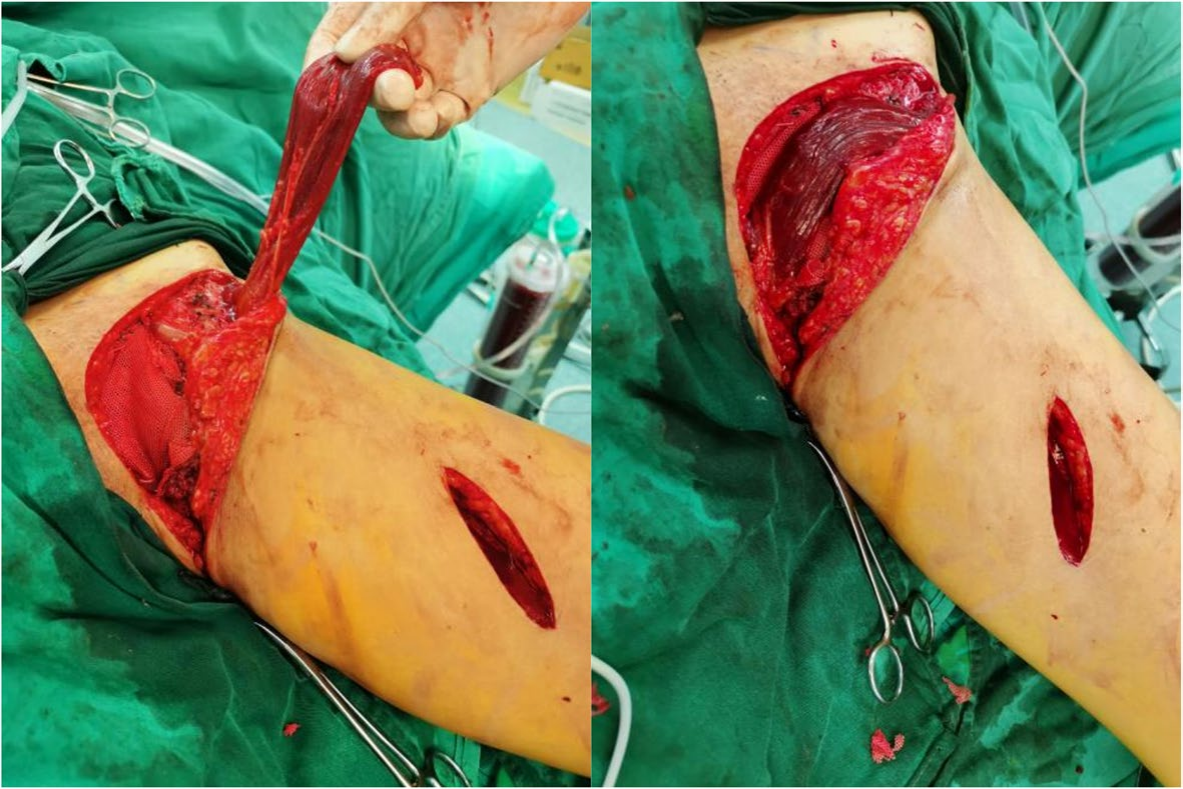
Intraoperative pictures show reconstruction of the pubic ring defect with mesh and the harvested pedicled sartorius flap (left, held up), and the mesh completely covered by pedicled sartorius flap (right)

Suture anchors were used and stabilized to the remaining bone and a mesh was sutured securely to the anchor to repair the pelvic ring defect. In order to harvest the sartorius muscle, a line originating from the anterior superior iliac spine to the proximal medial part of the tibia was drawn and an oblique incision of about 5 cm was made in the lower 1/3 of the line (Fig. 2). The sartorius muscle was detected in the incision immediately below the deep fascia and was cut off by the distal end of the incision. The free end of the sartorius was detached from the surrounding tissue and retracted to the pelvic incision through a subcutaneous tunnel. Special attention was paid to preserving the saphenous nerve and blood vessels supplying the proximal end of the sartorius muscle. The pedicled sartorius was rotated clockwise to cover the mesh and sutured to the surrounding tissue to fill the dead space left by tumor resection (Fig. 3). The wound was closed and prophylactic antibiotics were used within 24 h.

### Surgical-related factors and follow-up information

Of the 12 cases in the PSM group, the median duration of surgery was 130 min with a range of 120 to 150 min (Table 2), and median blood loss was 300 ml with a range of 200 to 450 ml. In the control group, the median duration of surgery was 122.5 min (range 90 to 150) and the median blood loss was 290 ml (range 200 to 400). There was no significant difference in the duration of surgery (*p* = 0.12) or blood loss (*p* = 0.51) between the two groups.

**Table 2:** Surgical-related factors and follow-up information

Surgical information	PSM group	Control group	
Duration of surgery (minutes, median, range)	130 (120–150)	122.5 (90–150)	*P* = 0.12
Blood loss (ml, median, range)	300 (200–450)	290 (200–400)	*P* = 0.51
Negative margin	12	8	
24 h prophylactic antibiotics used	12	8	
Bacteria detected	2	6	*P* = 0.03*
Wound dehiscence	1	5	*P* = 0.02*
Debridement	0	5	
Recurrence	0	0	
Herniation	0	6	
MSTS score (mean, range)	29 (27–30)	24 (22–26)	*P* < 0.05*

^*^P < 0.05

Negative surgical margins were achieved in all cases. Prophylactic antibiotics were administered within 24 h of the surgery in all cases as standard treatment.

In the PSM group, *Escherichia coli* was detected in the wound discharge in 2 cases and 1 patient developed wound dehiscence. Both cases recovered well after intravenous antibiotic treatment and no debridement was required. In the control group, 6 cases developed bacterial wound infection with either *Escherichia coli* or *Staphylococcus aureus*, and 5 cases developed wound dehiscence, each ultimately requiring at least one debridement. The control group showed a significantly higher rate of bacterial infection (*p* = 0.03) and wound dehiscence (*p* = 0.02) compared to the PSM group.

**Fig. 4:**
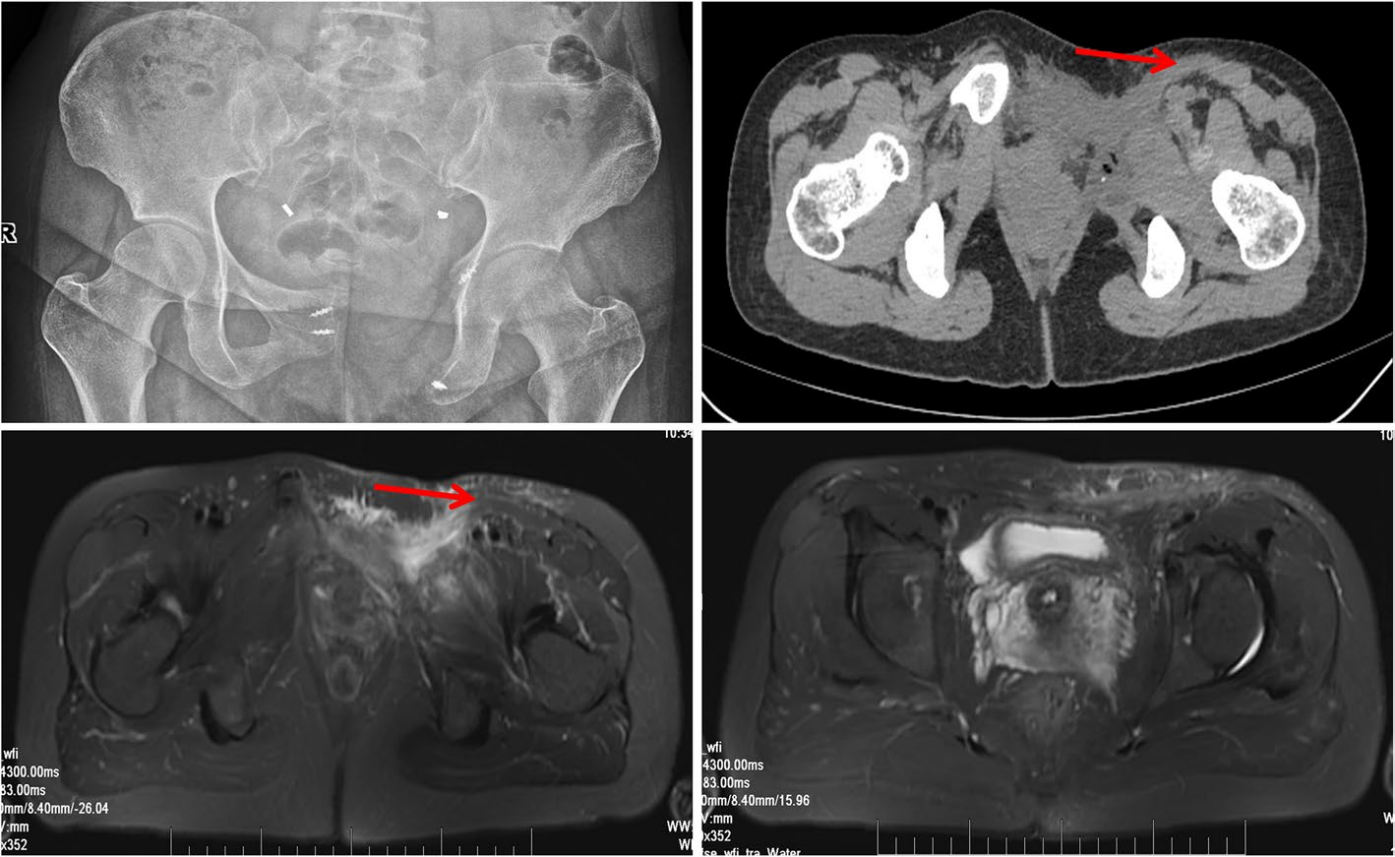
Pelvic bone imaging one year after surgery of case 3. X-ray (upper left) shows bony defect of pelvic ring after tumor resection. CT (upper right) and MRI scan (lower left) of the pelvic bone showing the pedicled sartorius flap as marked with red arrows. MRI scan of the pelvic bone (lower right) shows no sign of any herniation

Till the latest follow-up, no tumor recurrence was detected in either group. No sign of herniation was detected in the PSM group (Fig. 4) while herniation was found in 6 cases in the control group. All patients returned to independent activities of daily living. The PSM group had improved limb function with a median MSTS score of 29 (range 27–30), which was significantly higher (*p* < 0.001) than the control group which had a median MSTS score of 24 (range 22–26).

## Discussion

Wound complications such as infection and/or wound dehiscence rank among the most common complications associated with type III pelvic bone tumor resections. One study found that the rate of surgical site infection in pelvic bone tumor surgeries was 10.9% (33/302). This study proposed the use of implants and operative time exceeding 5 h as independent risk factors for infection [18]. In another study of 270 cases, 55 patients (20%) developed a deep infection. The infection rate was 15% in the patients without reconstruction and 26% in the patients with reconstruction. There were no significant differences in infection rates between construction with either allograft or metallic prostheses [19].

There are several potential explanations for the high rate of wound infections. Firstly, the pubic region is near the opening of the urinary and gastrointestinal tract, facilitating microbial seeding from these bacteria-laden areas and increasing the risk for infection [20]. Secondly, the resection of type III tumors without proper reconstruction results in large dead spaces that will become filled with static blood, providing a favorable environment for bacterial growth. Thirdly, the long and curved incisions required for proper tumor exposure and resection are more likely to compromise the blood supply and result in skin necrosis around the incision, increasing the risk of infection and the need for secondary debridement [21].

On top of wound infections, herniation of the bladder or other organs has also been reported following type III pelvic bone tumor resections [14, 22]. Multiple methods have been proposed to address postoperative herniation [14, 15, 23]. Allograft bones have been used to repair the pelvic ring defect following type III pelvic bone tumor resection with reportedly good results [15]. However, the use of allograft bones is limited by their high cost and low availability. Further, as discussed above, the high risk of infection discourages any attempts at pelvic ring reconstruction.

In this study, we found that the reconstruction of defects following type III pelvic tumor resection with the PSM technique resulted in significantly lower rates of perioperative complications. The PSM technique primarily involves two parts: the mesh and the pedicled sartorius flap. The mesh was employed to reconstruct the pelvic ring defect in the first step. Meshes have been widely and reliably used in clinics for decades to strengthen the inguinal region and deal with inguinal herniations. They are safe and easily available for most hospitals at very low cost. To address the dead space, we took advantage of the pedicled sartorius flap which was adjacent to the incision. The sartorius is the longest flap muscle in the body with segmental blood supplies [24], which enabled us to harvest the flap from either the proximal or distal end while retaining blood supply [24, 25]. The anatomic functions accomplished by the sartorius, such as flexion and rotation of the hip and knee joint, can be easily compensated for by the other muscles in the thigh. Since both points of insertions of the sartorius are palpable in the body with few anatomic variations, location, and harvesting of the sartorius flap according to the method described in this study is easily accomplished.

The novelty of this PSM technique lies in combining the mesh with the viable muscle flap. The mesh enabled rigid reconstruction of the bony defect, while the viable muscle flap filled the dead space and provided complete coverage of the mesh. This technique provided good functional outcomes in type III pelvic bone tumor surgeries, while being uncomplicated and inexpensive. In our cases, the pedicled sartorius flap easily covered the mesh and filled the dead space, likely contributing to the reduction in wound infections.

Still, the PSM technique should be utilized with caution. Firstly, special attention should be given to avoid placing the suture anchor into the hip joint. Secondly, care should be taken to preserve as much of the vasculature supplying the sartorius muscle as possible. Impairing the blood supply to the flap would compromise its viability. Finally, one disadvantage of the PSM technique is the requirement of an additional incision in the lower medial thigh to harvest the flap.

This study is not without its limitations. One major shortcoming is the relatively small sample size of this study. In addition, the inclusion of elderly patients may have introduced unknown factors that may have affected wound healing and complications. A future study that includes a larger sample size and a greater number of patients under the age of 65 would strengthen the validity of our findings so far.

## Conclusions

The pedicled sartorius flap combined with mesh (PSM) technique may reduce the rate of wound complications and herniations following type III pelvic bone tumor resections.

## Data Availability

All data generated or analyzed during this study are included in this published article.

## Abbreviations

PSM: Pedicled sartorius flap and mesh
MSTS: Musculoskeletal Tumor Society
CT: Computed tomography
MRI: Magnetic resonance imaging
